# Role of Mitochondria in Regulating Lutein and Chlorophyll Biosynthesis in *Chlorella pyrenoidosa* under Heterotrophic Conditions

**DOI:** 10.3390/md16100354

**Published:** 2018-09-28

**Authors:** Zhi-hui Liu, Tao Li, Qing-yu He, Zheng Sun, Yue Jiang

**Affiliations:** 1 Key Laboratory of Exploration and Utilization of Aquatic Genetic Resources, Ministry of Education, Shanghai Ocean University, Shanghai 201306, China; m160150388@st.shou.edu.cn; 2 International Research Center for Marine Biosciences, Ministry of Science and Technology, Shanghai Ocean University, Shanghai 201306, China; 3 National Demonstration Center for Experimental Fisheries Science Education, Shanghai Ocean University, Shanghai 201306, China; 4 College of Basic Science, Tianjin Agricultural University, Tianjin 300384, China; tli@tjau.edu.cn; 5 College of Life Science and Technology, Jinan University, Guangzhou 510632, China; tqyhe@jnu.edu.cn; 6 Runke Bioengineering Co. Ltd., Zhangzhou, Fujian 363503, China

**Keywords:** mitochondrial respiratory electron transport chain, mitochondrial retrograde regulation, redox state, energy metabolism, proteomics

## Abstract

The green alga *Chlorella pyrenoidosa* can accumulate lutein and chlorophyll under heterotrophic conditions. We propose that the mitochondrial respiratory electron transport chain (mRET) may be involved in this process. To verify this hypothesis, algal cells were treated with different mRET inhibitors. The biosynthesis of lutein and chlorophyll was found to be significantly stimulated by salicylhydroxamic acid (SHAM), whereas their contents substantially decreased after treatment with antimycin A and sodium azide (NaN_3_). Proteomic studies revealed profound protein alterations related to the redox and energy states, and a network was proposed: The up-regulation of peroxiredoxin reduces oxidized glutathione (GSSG) to reduced glutathione (GSH); phosphoenolpyruvate carboxykinase (PEPCK) catalyzes the conversion of oxaloacetic acid to phosphoenolpyruvate, and after entering the methylerythritol phosphate (MEP) pathway, 4-hydroxy-3-methylbut-2-en-1yl diphosphate synthase reduces 2-*C*-methyl-d-erythritol-2,4-cyclodiphosphate (ME-Cpp) to 1-hydroxy-2-methyl-2-(E)-butenyl 4-diphosphate (HMBPP), which is closely related to the synthesis of lutein; and coproporphyrinogen III oxidase and ChlI play important roles in the chlorophyll biosynthetic pathway. These results supported that for the heterotrophic *C. pyrenoidosa*, the signaling, oriented from mRET, may regulate the nuclear genes encoding the enzymes involved in photosynthetic pigment biosynthesis.

## 1. Introduction

Microalgae can utilize organic carbon as a carbon and energy source to grow and reproduce without relying on light, which is known as heterotrophic growth [1]. The advantages of heterotrophic cultivation include high density, high degree of process control, consistent and reproducible biomass yields, and so on; however, not all microalgae can be heterotrophically cultivated. Significant progress in the heterotrophic cultivation of *Chlorella* has been made in terms of the cultivation modes and yield, and furthermore, commercial successes have been achieved [2]. It was discovered that *Chlorella pyrenoidosa* not only accumulates up to 150 g/L of biomass under heterotrophic conditions [3], but can also synthesize lutein and chlorophyll [4,5]. Lutein is a member of carotenoids, which represent the most common group of pigments in marine environments. Lutein can preferentially cross the blood–brain barrier and accumulate in brain tissue, thus playing positive roles in neurocognitive functioning [6]. Besides, lutein is also well known to ameliorate cataract, age-related macular degeneration and other optical diseases [7,8,9]. As there is no photosynthesis that occurs during the heterotrophic cultivation, the synthesis of photosynthetic pigments is supposed to be hampered accordingly. Therefore, if the nuclear genes that encode the enzymes involved in photosynthetic pigment biosynthesis and chloroplast formation are not activated by other factors or organelles, the amounts of synthesized chloroplasts and pigments (lutein and chlorophyll) will gradually decrease and disappear as cell division progresses. In recent years, studies have shown that the mitochondria not only act as an energy provider, but also have retrograde regulation of the development of other organelles (such as chloroplasts) in green algae and plants [10,11,12]. The mitochondrial respiratory electron transport chain (mRET) is a key regulator in this process. Several studies have shown that mRET plays a crucial role in the retrograde regulation of the expression of photosynthetic genes, in some microorganisms, under dark conditions. Matsuo and Obokata [11] reported that under heterotrophic conditions, the photosynthetic electron transport (PET) signals and RET signals in *Chlamydomonas reinhardtii* induced the expression of nuclear photosynthetic genes. Li et al. [12] also reported that the mRET of *Chlorella zofingiensis* was able to initiate signal transduction and ultimately induced the expression of photosynthesis-related genes, such as those associated with chloroplast formation and pigment biosynthesis. Based on the above information, we hypothesized that the accumulation of photosynthetic pigments in *C. pyrenoidosa* under heterotrophic conditions may be related to the regulation of mRET. To verify this hypothesis, heterotrophic algal cells were treated with different mRET inhibitors, namely salicylhydroxamic acid (SHAM), antimycin A, and sodium azide (NaN_3_), and proteomics analysis was conducted. A method coupling two-dimensional electrophoresis (2-DE) with matrix assisted laser desorption-time-of-flight/time-of-flight tandem mass spectrometry (MALDI-TOF/TOF MS/MS) was used in this study. The findings may contribute to the knowledge of proteomics of marine natural products.

## 2. Results

### 2.1. Variation in Lutein and Chlorophyll Biosynthesis

**Figure 1:**
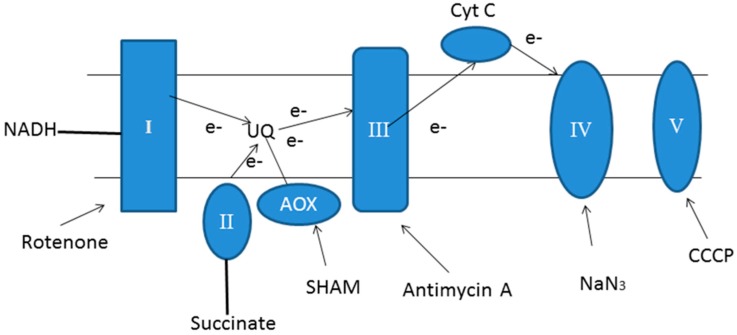
Schematic diagram of the mitochondrial respiratory electron transport chain (mRET) and the action sites of inhibitors. Complex I, reduced nicotinamide adenine dinucleotide (NADH) coenzyme Q reductase; complex II, succinate dehydrogenase; UQ, ubiquinone; complex III, cytochrome bc1 complex; complex IV, cytochrome c oxidase; complex V, ATPase; AOX, alternative oxidase; Cyt c, cytochrome c; SHAM, salicylhydroxamic acid; CCCP, carbonyl cyanide m-chlorophenylhydrazone.

In this study, we investigated the role of mRET in the retrograde regulation of lutein and chlorophyll biosynthesis in *C. pyrenoidosa* under heterotrophic conditions via mitochondrial dysfunction induced by specific inhibitors. The reported action sites of those inhibitors are shown in Figure 1. SHAM inhibited the alternative pathway by inactivating alternative oxidase (AOX) in mRET, while antimycin A blocked the cytochrome oxidase pathway by interrupting the electron transport from cytochrome b to c [13]. NaN_3_ effectively inactivated the cytochrome oxidase of mRET [14]. Rotenone inhibited the respiratory complex I, and carbonyl cyanide m-chlorophenylhydrazone (CCCP), an oxidative phosphorylation uncoupler, suppressed the ATP synthesis [15]. For each inhibitor, various concentrations were tested at first (rotenone: 0.01–0.1 mM, SHAM: 0.2–2.0 mM, CCCP: 0.01–0.1 mM, antimycin A: 0.05–0.5 mM, and NaN_3_: 0.01–0.1 mM). After evaluation, the optimal concentrations were determined for each inhibitor (data not shown), and were used for subsequent experiments.

As shown in Table 1, the biomass contents of *C. pyrenoidosa* significantly decreased after treatment with all inhibitors. With respect to pigments, treatment with antimycin A (0.1 mM) and NaN_3_ (0.05 mM) induced a sharp decrease in lutein and chlorophyll contents. On the other hand, the accumulation of lutein and chlorophyll was enhanced by the treatment with SHAM (0.5 mM). The effects of CCCP (0.032 mM) and rotenone (0.032 mM) were not so significant compared with those three inhibitors, suggesting that complexes І and V were less involved in the photosynthetic pigment biosynthesis.

### 2.2. Analysis of Redox and Energy States

**Figure 2:**
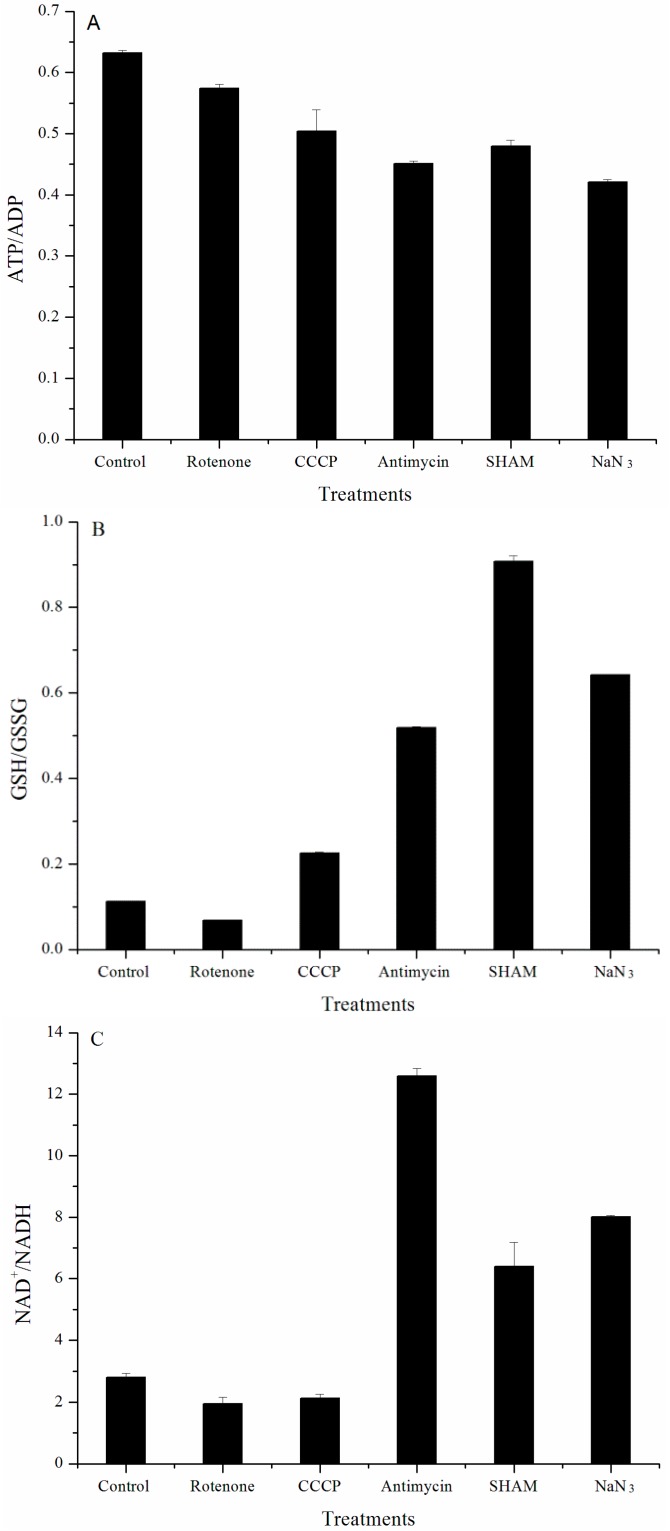
Variation in energy and redox states in *Chlorella pyrenoidosa* after treatment with mRET inhibitors. (**A**) ATP/ADP; (**B**) GSH/GSSG; (**C**) NAD^+^/NADH. Data are expressed as averages ± S.D. of three independent measurements.

Cellular energy and redox states analysis showed that mitochondrial dysfunction caused a decrease in the ATP/ADP ratio (Figure 2A). The ratio of GSH/GSSG (Figure 2B) and NAD^+^/NADH (Figure 2C) became significantly higher after treating the cells with SHAM (0.5 mM), NaN_3_ (0.05 mM), and antimycin A (0.1 mM). For CCCP (0.032 mM) and rotenone (0.032 mM), their effects were much less prominent. Taken together, only cells treated with SHAM, NaN_3_, and antimycin A were further examined in the following proteomic studies.

### 2.3. SDS-PAGE of Total Proteins

**Figure 3:**
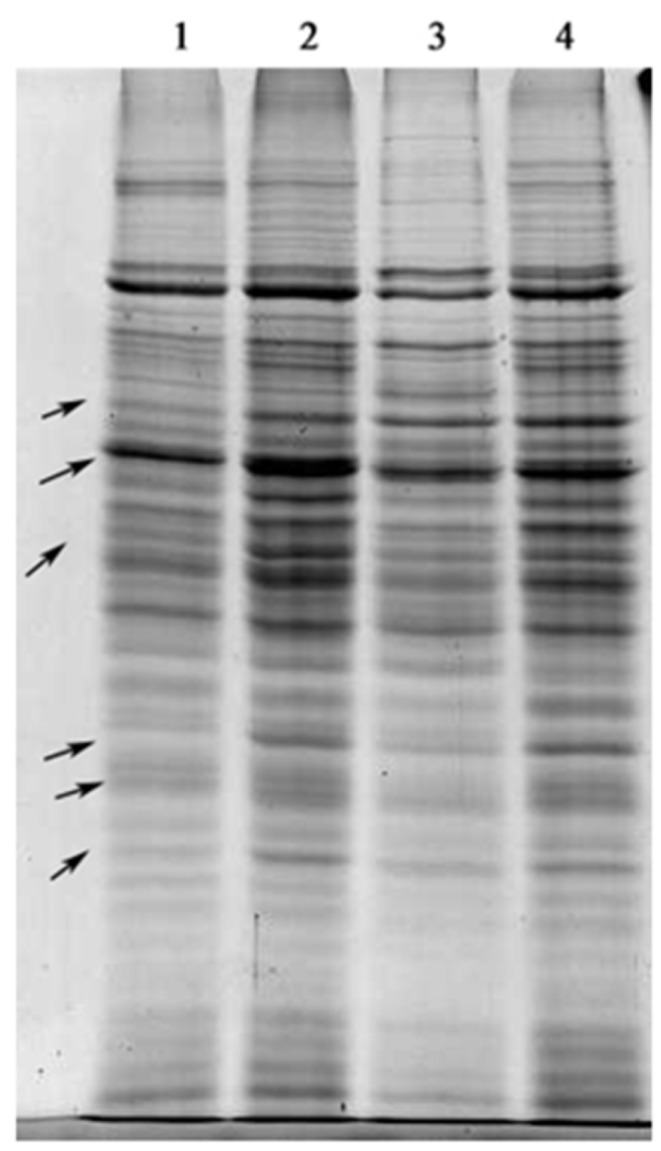
Total proteins from mRET inhibitor-treated *C. pyrenoidosa* and the control. Lane 1, antimycin A treatment; lane 2, SHAM treatment; lane 3, NaN_3_ treatment; lane 4, control. Arrows: Representative bands in lanes 1–3 exhibiting significant difference with lane 4. The samples were loaded at the same protein concentration.

Total soluble proteins from algal cells, that were treated with antimycin A, SHAM and NaN_3,_ were extracted and compared. Figure 3 shows that the protein constitutions between mRET inhibitor-treated and untreated algal cells changed significantly, particularly the bands indicated by the arrows. It can thus be concluded that numerous physiological reactions occurred following mitochondrial dysfunction in dark-grown *C. pyrenoidosa*.

### 2.4. 2-DE Analysis and Identification of Differentially Expressed Proteins

**Figure 4:**
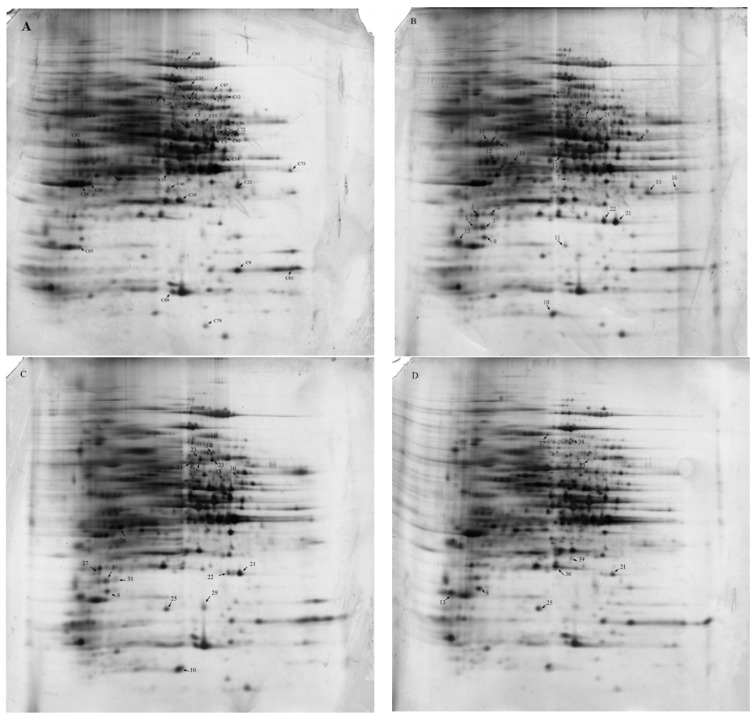
2-D gel images of total proteins from mRET inhibitor-treated *C. pyrenoidosa* and the control. The abundantly expressed spots, marked in the images with an arrow, were selected for identification. They are listed in Table 2 and Table 3. (**A**) control; (**B**) SHAM treatment; (**C**) antimycin A treatment; (**D**) NaN_3_ treatment. 2-DE was performed by loading 150 μg protein in a 13-cm non-linear immobilized pH gradient (IPG) strip with a p*I* range of 3–10, followed by separation on 12.5% SDS-PAGE after 1-D isoelectric focusing (IEF).

Comparative proteomics was employed to examine the variation of protein expression after mitochondrial dysfunction. The extracted proteins were analyzed using 2-DE and the protein expression patterns from the three independent experiments showed high reproducibility. The successfully identified proteins were marked in the gel images, shown in Figure 4A–D. Further, detailed information is provided in Table 2 and Table 3. Compared to the control group, a total of 38 proteins (24 up-regulated and 14 down-regulated) were successfully identified (1.5-fold, *P* ˂ 0.05) in the SHAM-treated algal cells (Table 2 and Table 3). A total of 36 proteins (16 up-regulated and 20 down-regulated) and 31 proteins (10 up-regulated and 21 down-regulated) were differentially expressed (1.5-fold, *P* ˂ 0.05) in algal cells treated with antimycin A and NaN_3_ (Table 2 and Table 3), respectively.

### 2.5. Functional Categorization

The differentially expressed proteins were then categorized. As no functional classification is available for *C. pyrenoidosa*, the categorization performed in this study was based on the genome of another member of the same genus, namely *C. variabilis*. The top candidate with a score higher than the threshold value (*P* < 0.05) was considered positive. If negative, searching against the NCBI Nr or Swiss-Prot database was also performed. Additionally, de novo sequencing and MS-BLAST searches were also employed as an identification strategy in this study. Those identities together with the p*I*, molecular weight, reported functions, and fold-change in comparison to the control are listed in Table 2 and Table 3. Among all the identities listed in Table 2 and Table 3, most of the protein orthologues belonged to *C. variabilis*, while some proteins also belonged to other algae, such as *C. vulgaris*, *Parachlorella kessleri*, *Chlamydomonas reinhardtii*, or plants, such as *Arabidopsis thaliana*. 

According to the identification results, it was found that the protein names of *C. variabilis* were almost all “hypothetical”. This is because most of the genome annotation of *C. variabilis* is still in progress. Fortunately, the Gene Ontology (GO) or EuKaryotic Orthologous Groups (KOG) annotations on the majority of the proteins in *C. variabilis* have been completed and are available online. Therefore, in this study, these GO annotations were used to interpret the function of the identified hypothetical proteins. The functional categorization of abundantly expressed proteins after treatment with three mRET inhibitors was shown in Figure S1.

## 3. Discussion

The green algae *Chlorella* can be grown both autotrophically and heterotrophically. Keeping a portion of pigments under the dark condition is a self-adaptive strategy, which may help the algae quickly adapt to the light when the culture is shifted to the phototrophic condition. From the perspective of evolution, this may be regarded as a transition state between eukaryotic microorganisms (100% heterotrophy) and green plants (100% phototrophy). In this study, the dysfunction of mRET led to changes in the contents of lutein and chlorophyll in *C. pyrenoidosa* under heterotrophic conditions. Lutein and chlorophyll biosynthesis were dramatically stimulated after treatment with SHAM, while their contents considerably decreased after treatment with antimycin A and NaN_3_. This suggests that mRET participates in the synthesis of photosynthetic pigments, which is consistent with previous reports [11]. Proteomic analysis revealed a large number of differentially expressed proteins after the dysfunction of mRET (Table 2 and Table 3). Among them, some representative proteins were discussed in below to interpret the mechanism. These proteins were divided into four groups based on the functional classification, namely antioxidant proteins, chloroplast proteins, transcription and protein fate-related proteins, and metabolism and energy-related proteins. In each group, the proteins have direct or indirect relation to pigment synthesis. 

### 3.1. Up-Regulation of Antioxidant Proteins

As a result of the excess energy present in the mitochondria, which easily reduces oxygen, mitochondrial reactive oxygen species (mtROS) are often unavoidably produced during the aerobic respiration process, even under normal conditions [16]. Thus, mtROS are even more likely to be produced under abiotic or biotic stress conditions. The study of Tang et al. [17] indicated that mtROS could directly affect the functionality of mRET complexes by oxidizing their iron–sulfur centers, thus aggravating ROS production. Previous studies have confirmed that mRET dysfunction induced by specific inhibitors, such as SHAM and antimycin A, can greatly enhance mtROS production. In response, cells activate multiple defense systems to detoxify these excessive harmful mtROS and protect the cellular organelles [18,19,20]. Our findings corroborated our expectations, particularly in the SHAM treatment. Several antioxidant enzymes, including peroxiredoxin TSA1 (spot U3), 2-Cys peroxiredoxin (spot U5) and hypothetical protein (spot U7), exhibited antioxidant features according to the GO analysis and were all up-regulated. Peroxiredoxin TSA1 (spot U3) and superoxide dismutase (SOD) (spot U36) were also up-regulated in antimycin A- and NaN_3_-treated cells (Table 3). Peroxiredoxin is an antioxidant protein with an activity site of cysteine that is oxidized by a peroxide substrate. The oxidized peroxiredoxin can be reduced by GSH [21]. Therefore, the proteomic results in this study could explain the changes in redox state following these treatments (NaN_3_, antimycin A, and SHAM). In addition, the hydroxyl radicals in ROS react directly with DNA components, destroying bases and DNA backbones. Additionally, mtROS, such as H_2_O_2_, can also penetrate the mitochondrial membrane and enter into the cytoplasm [22,23]. This results in the further recruitment of GSH molecules to reduce the oxidized antioxidants, such as peroxiredoxin and ascorbate. It is thus reasonable to expect that the ratio of GSH/GSSG would increase. The study of Foyer and Noctor [24] also suggested that GSH is a key arbiter of intracellular redox potential, and ascorbate plays an important role in setting thresholds for cytoplasmic signaling. In addition to being a harmful oxidant, mtROS is also widely believed to participate in mitochondria to nucleus signaling—that is, mitochondrial retrograde regulation [16,18,25,26]. Ho et al. [27] showed that redox homeostasis was disturbed in nicotinamide nucleotide transhydrogenase-deficient cells, resulting in increased mtROS generation and the eventual regulation of the mitochondrial retrograde signaling response. Some researchers have indicated that mtROS, as secondary signaling messengers, lead to the expression of nuclear genes after leaving the mitochondria [18,28]. However, Matsuo and Obokata [11] suggested that mtROS were not responsible for nuclear-located photosynthetic gene expression as a result of increased ROS production following treatment with three inhibiters in *Chlamydomonas reinhardtii*, although antimycin A and potassium cyanide decreased gene expression, and SHAM increased gene expression. This implies that mtROS might not be the only explanation for the variation in redox state. In addition, chloroplast thioredoxin peroxidase (spot U1) was up-regulated after treatment with SHAM (Table 3). This indicated that messengers from the mitochondria, such as mtROS, interacted with the chloroplast in *C. pyrenoidosa* and caused a change in the redox state in the chloroplast after the induction of mitochondrial dysfunction by SHAM.

### 3.2. Variation in Chloroplast Protein Expression Following Mitochondrial Dysfunction

In general, cellular components and organelles are thought not to be isolated and independent, but rather interact and communicate with each other frequently and in a complex manner. Very few studies have focused on cellular organelle communication in green algae, especially in non-model algae. In this study, several chloroplast proteins were found to be up- or down-regulated after inhibitor treatment (Table 2 and Table 3). For example, chloroplast ribosome protein L22 (spot U19) was up-regulated after treatment with SHAM, while chloroplast 30 S ribosome protein S4 (spot U25) was up-regulated in both antimycin A- and NaN_3_-treated cells. 

Ribosome protein L22 was found to be the main component of chloroplast 50S ribosome and is nuclear-encoded in the green alga *C. reinhardtii* [29]. It could thus be assumed that the retrograde signal from the mitochondria directly or indirectly activated chloroplastic gene expression and protein translation in the chloroplast to adapt to the new cellular environment. This perspective is supported by the observation that chloroplastic tRNA (Ile)-lysidine synthase, which changes amino acids specificity from methionine to isoleucine, was also up-regulated after SHAM treatment. The expression level of other proteins involved in the chlorophyll and lutein biosynthetic pathways in the chloroplast was also altered. Coproporphyrinogen III oxidase catalyzes the biosynthesis of protoporphyrinogen IX, which is converted into protoporphyrin IX; a precursor for chlorophyll and heme biosynthesis in two branch pathways [30,31]. In this study, coproporphyrinogen III oxidase (spot D14, Table 3) in both antimycin A- and NaN_3_-treated algal cells was down-regulated. This could possibly account for the decrease in chlorophyll content following these two treatments. However, the coproporphyrinogen III oxidase level also significantly decreased after SHAM treatment, which enhanced chlorophyll production markedly. This may be closely related to the up-regulated expression of another enzyme, the magnesium chelatase subunit of protochlorophyllide reductase (ChlI) (spot U4), which is a key enzyme involved in the chlorophyll biosynthesis branch pathway. In contrast, the expression level of ChlI was down-regulated in antimycin A- and NaN_3_-treated algal cells (Table 3). It is thus reasonable to assume that the up-regulated protochlorophyllide reductase captured more protoporphyrin IX for chlorophyll biosynthesis, even though the pool of protoporphyrinogen IX decreased, due to the down-regulation of coproporphyrinogen III oxidase. This might account for the observation that coproporphyrinogen III oxidase was down-regulated following SHAM treatment, while lutein and chlorophyll biosynthesis increased.

Previous studies have shown that the intermediate of chlorophyll biosynthesis participates in the chloroplast retrograde regulation signaling pathway. Currently, the most studied intermediate is Mg-protoporphyrin IX monomethyl ester, the accumulation of which greatly represses the expression of nuclear photosystem II (Lhcb) mRNA or other chloroplast genes in *C. reinhardtii* and other organisms [32,33,34,35]. In addition, Mg-chelatase, composed of subunits ChlD, ChlH, and ChlI, also participates in the plastid to nucleus signal transduction pathway in *Arabidopsis,* with the exception of the function of inserting Mg^2+^ into the porphyrin ring of Proto IX [36,37]. Mochizuki et al. [36] proposed that ChlH not only serves as chelatase in tetrapyrrole biosynthesis, but functions as a tetrapyrrole sensor in the chloroplast to nucleus signaling pathway. ChlH also possibly participates in the plastid to nucleus signaling pathway by forming ChlH-tetrapyrrole complexes with other cofactors [31,38,39,40].

All previous experiments have only interpreted the negative aspect of the chloroplast retrograde signal transduction pathway whereby it represses nuclear-encoded chloroplastic gene expression. However, the positive aspects of the signaling pathway have not been evaluated. If chlorophyll biosynthesis is improved, the synthesis of more chloroplast proteins (e.g., light-harvesting complex II) would be required to provide sufficient binding locations for these newly synthesized pigments. We thus proposed the hypothesis that the magnesium chelatase subunit also serves as a sensor, or directly participates in the plastid to nucleus signaling transduction pathway, which activates chloroplast genes expression when chlorophyll biosynthesis is enhanced. In fact, another enzyme, 4-hydroxy-3-methylbut-2-en-1yl diphosphate synthase (ispG) (spot U21), was greatly up-regulated after SHAM treatment (Table 3). Furthermore, it is the same group of enzyme as 1-hydroxy-2-methyl-2-(E)-butenyl 4-diphosphate (HMBPP) reductase, which plays rate-determining roles in controlling chloroplastic methylerythritol phosphate (MEP) pathway fluxes towards the biosynthesis of isoprenoids and carotenoids in plants [41,42]. Moreover, the expression level of ispG was also up-regulated to some extent after antimycin A and NaN_3_ treatment (Table 3).

### 3.3. Variation in Transcription and Protein Fate-Related Protein Expression

The results in Table 2 and Table 3 show that some transcription and protein fate-related proteins were successfully identified. Among the identified proteins, phytochrome A, 14-3-3-like protein, and TCP-1 eta subunit (CCT7) were the most striking, because of their special functions in plants. Phytochrome A (spot D25) and TCP-1 eta subunit (CCT7) (spot D30) were both down-regulated in NaN_3_- and antimycin A-treated *C. pyrenoidosa*, whereas the expression level of these two proteins did not change after treatment with SHAM (Table 3). A 14-3-3-like protein (spot D54) was down-regulated after treatment with antimycin A, while no changes were observed in NaN_3_- and SHAM-treated cells. 

In plants and green algae, adjustments or adaptions to variations in the light irradiation environment are vitally important for growth and development, and this process is accomplished through a network of photoreceptors. Among these photoreceptors, phytochromes, which are able to absorb red and far-red light, are the most thoroughly studied to date [43]. Previous studies showed that phytochrome A was responsible for the regulation of light-inducible photosynthetic genes, such as genes encoding chlorophyll a/b binding proteins, small subunit of ribulose 1,5-bisphosphate carboxylase, pchlide oxidoreductase A, and phytoene synthase [44,45,46]. Chlorophyll a/b-binding proteins primarily associated with chlorophyll and lutein constitute the apoproteins of the light-harvesting complex of photosystem II [47]. In this study, it was observed that the expression level of phytochrome A increased when the algal cells were treated with SHAM. Simultaneously, the content of chlorophyll and lutein also increased. When treated with antimycin A and NaN_3_, the expression level of phytochrome A decreased, and the contents of chlorophyll and lutein also decreased. It could be concluded that the regulatory process might be related to the expression level of the chlorophyll a/b binding protein encoded by the photosynthetic gene. Phytochromes are typically synthesized under dark conditions, in a red light absorbing form, Pr—which transforms into the biologically active form Pfr after absorbing far-red light [48]. Afterwards, the active Pfr is translocated into the nucleus where it interacts with a series of signaling components and ultimately activates the expression of photomorphogenesis-related genes. Some of these signaling components were reported to be phytochrome-interacting factors (PIFs), which are responsible for the repression of photomorphogenesis in darkness in *Arabidopsis* [49]. Whereas in the light, phytochromes either promote the degradation of PIFs or inhibit their binding to their target promoters [50,51]. Toledo-Ortiz et al. [48] indicated that the degradation of a high level of cellular PIF following interaction with the bioactive form Pfr was mainly responsible for the rapid accumulation of carotenoids in coordination with increased chlorophyll biosynthesis and chloroplast protein formation. PIFs are a subfamily of basic-helix-loop-helix transcription factors, which have been found in most plants and some green algae, such as *C. vulgaris* [52]. It is thus reasonable to assume that PIFs are also present in *C. pyrenoidosa*, even though no related reports exist. Therefore, we speculated that small amounts of phytochrome A were activated through another way in *C. pyrenoidosa* under dark conditions, and the reaction of Pfr with PIFs allowed small amounts of chlorophyll and carotenoids to be synthesized in *C. pyrenoidosa* grown in the dark. It is thus sensible that when the level of phytochrome A decreased following treatment with NaN_3_ and antimycin A, that less chlorophyll and lutein would be synthesized in *C. pyrenoidosa*. Phytochromes have two major domains: One is an amino-terminal domain carrying a tetrapyrrole chromophore, which is used for absorbing red or far-red light, and the other is a carboxyl-terminal domain used for binding with other signaling components [43]. The currently accepted mechanism for the activation of the phytochrome system is that the conformation of the phytochrome system changes and becomes active for other protein-binding activities after absorbing light. Therefore, if other chaperonins bind with phytochrome and the protein conformation alters appropriately, then phytochrome can also be activated similarly. Mummert et al. [53] reported that chaperonin TCP-1 could be copurified with phytochrome and help refold the inactive phytochrome to the photoactive form. Su and Lagarias [54] also discovered the light-independent phytochrome signaling pathway in an *Arabidopsis* mutant. In this study, we also found that the expression level of chaperonin TCP-1 and phytochrome A simultaneously decreased after treatment with antimycin A and NaN_3_. We thus speculated that chaperonin TCP-1 might possibly also facilitate the refolding of phytochrome A in *C. pyrenoidosa* grown in the dark. However, this requires further verification in future studies. Another chaperonin 14-3-3 protein, which has multifunctional phosphoserine binding sites, participates in many important cellular processes. In higher plants, 14-3-3 proteins are phosphoserine-binding proteins that regulate the activities of a wide array of targets through protein-protein interactions [55]. Kinoshita et al. [56] reported that 14-3-3 interacted with phototropins, another photoreceptor, in a phosphorylation-dependent manner and could be important in phototropin-mediated responses. In our experiment, we also found that a 14-3-3 like protein (Spot D54) was down-regulated 1.8-fold (Table 3) after antimycin A treatment, but not in NaN_3_-treated cells. Many 14-3-3 protein species have been reported in plants, such as *Arabidopsis* [57], rice [58], and tomato [59] where they regulate numerous cellular processes via interaction with their target proteins in a phosphorylation-dependent manner. Hong et al. [57] suggested that AtSKIP31 regulates primary root growth under nitrogen deficiency by degrading *Arabidopsis* 14-3-3s, which are related to cell growth. 

### 3.4. Variation in Metabolism and Energy-Related Protein Expression

The results in Table 2 and Table 3 show that the identified proteins related to metabolism and energy production accounted for a large proportion of the differentially expressed proteins. Among them, d-3-phosphoglycerate dehydrogenase (spot D17), a key enzyme involved in glycolysis, was simultaneously down-regulated after the three treatments (Table 3). 

Once the induction of mitochondrial dysfunction by the inhibitors occurs, the TCA cycle and glycolysis process would definitely be influenced. The expression levels of other enzymes related to amino acid biosynthesis or nitrate metabolism also changed, such as phosphoserine aminotransferase, which is involved in serine biosynthesis and was down-regulated in SHAM-treated cells, while *N*-(5′-phosphoribosyl) anthranilate isomerase, which participates in tryptophan biosynthesis, was up-regulated in SHAM-treated cells. Among the identified proteins, the identification of phosphoenolpyruvate carboxykinase (PEPCK) (spot D37) was most significant in this study. This enzyme was found to be down-regulated in antimycin A- and NaN_3_-treated cells, but not in SHAM-treated cells (Table 3). 

**Figure 5:**
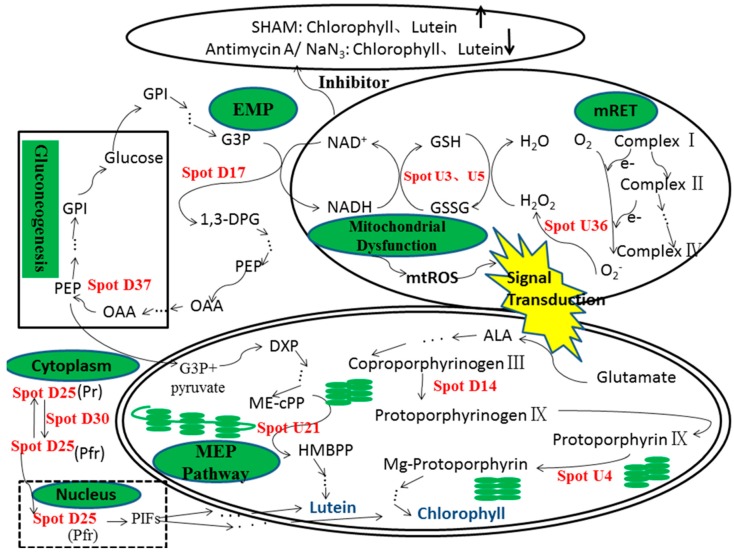
An overview of the metabolic pathways of *C. pyrenoidosa* and the interactions between the identified up- or down-regulated proteins. Antioxidant proteins are displayed in the mitochondria. The chloroplast proteins are displayed in chlorophyll and lutein biosynthesis. Transcription and protein fate-related proteins are displayed in the signaling from cytoplasm to nucleus. Metabolism and energy-related proteins are displayed in glycolysis and gluconeogenesis pathways. ALA, 5-aminolevulinic acid; DXP, 1-deoxy-d-xylulose 5-phosphate; 1,3-DPG, 1,3-diphosphoglycerate; EMP, glycolytic pathway; GPI, Glucose-6-phosphate; G3P, glyceraldehyde-3-phosphate; HMBPP, 1-hydroxy-2-methyl-2-(E)-butenyl 4-diphosphate; ME-cPP, 2-*C*-methyl-d-erythritol-2,4-cyclodiphosphate; mRET, mitochondrial respiratory electron transport chain; MEP Pathway, methylerythritol phosphate pathway; OAA, oxaloacetic acid; PEP, phosphoenolpyruvate; PIF, phytochrome-interacting factor; Pfr, active form; Spot U3, peroxiredoxin TSA1; Spot U4, Magnesium chelatase subunit of protochlorophyllide reductase (ChlI); Spot U5, 2-Cys peroxiredoxin; Spot D14, coproporphyrinogen III oxidase; Spot D17, d-3-phosphoglycerate dehydrogenase; Spot U21, 4-hydroxy-3-methylbut-2-en-1yl diphosphate synthase (ispG); Spot D25, Phytochrome A; Spot D30, TCP-1 eta subunit (CCT7); Spot U36, Superoxide dismutase; Spot D37, phosphoenolpyruvate carboxykinase (PEPCK). The solid line box indicates that the metabolic pathway has been verified, and the dashed box indicates that it needs to be verified. The “→” indicates that the reaction is completed in one step, and the “→∙∙∙→” indicates that the multi-step reactions are required.

PEPCK, also referred to as ATP-oxaloacetate carboxylase, catalyzes the formation of phosphoenolpyruvate (PEP) [60]. PEP is usually synthesized in the mitochondria and transported into plastids as the precursor for the shikimate pathway, which provides aromatic compounds for plastid development in plants [61]. In plants, the translocation of PEP is vital for plastid development and the biosynthesis of carotenoids and chlorophylls. In addition, carotenoid biosynthesis is widely accepted to occur, via the MEP pathway, in the plastids of plants and green algae, by using pyruvate and glyceraldehyde-3-phosphate as precursors [62]. Huang et al. [63] also reported that the biosynthesis of isopentenyl diphosphate by the MEP pathway was found to be correlated with carotenoid biosynthesis in *C. zofingiensis*. Pyruvate is directly converted from PEP by pyruvate kinase. In gluconeogenesis, PEPCK catalyzes the reversible decarboxylation of oxaloacetate to PEP and carbon dioxide. The study of Wu et al. [64] showed that the abundance of PEPCK was increased as peach fruits reached the ripening stage, and then gradually declined at the senescence stage, indicating that PEPCK might play an important role in maintaining normal levels of glucose through gluconeogenesis, especially when a large amount of glucose is consumed to provide energy. Thus, if the supply of PEP is deficient, carotenoids and chlorophyll biosynthesis are definitely affected. This suggests that the down-regulation of PEPCK is possibly responsible for the decrease in both lutein and chlorophyll accumulation in antimycin A- and NaN_3_-treated *C. pyrenoidosa*. Further analysis is required to reveal the details of this mechanism. An overview of the metabolic pathways of *C. pyrenoidosa* and the interactions between the identified up- or down-regulated proteins is provided in Figure 5. Mitochondrial dysfunction leads to changes in the intracellular redox state and energy metabolism. Among these changes, up-regulated peroxiredoxin reduces GSSG to GSH, and in the glycolytic pathway, d-3-phosphoglycerate dehydrogenase, which oxidizes glyceraldehyde 3-phosphate to 1,3-diphosphoglyceric acid, is down-regulated. At this moment, the amount of NADH produced is correspondingly reduced, which can explain the increases in the ratios of NAD^+^/NADH and GSH/GSSG. In addition, in the gluconeogenesis pathway, PEPCK catalyzes the conversion of oxaloacetic acid to PEP. After entering the MEP pathway, ispG reduces 2-*C*-methyl-d-erythritol-2,4-cyclodiphosphate (ME-cPP) to HMBPP, which is closely related to the synthesis of lutein. And coproporphyrinogen III oxidase and ChlI play important roles in the chlorophyll biosynthetic pathway. 

## 4. Materials and Methods

### 4.1. Microalga and Culture Conditions

The algal strain *C. pyrenoidosa* was purchased from Carolina Biological Supply Co., Burlington, NJ, USA. The basic medium was the same as that used in a previous report, and the potassium concentration was 1.25 g/L [4]. The algal cells were inoculated into 250-mL Erlenmeyer flasks containing 100 mL medium at an inoculation volume of 10% (*v*/*v*). Heterotrophic cultivation was achieved by culturing the algal cells in a basic medium supplemented with 40 g/L glucose and 8.25 g/L potassium nitrate at 28 °C with orbital shaking at 180 rpm under darkness. For biomass measurement, a 15 mL culture suspension was filtered through a pre-dried Whatman GF/C filter paper (1.6 μm pore size) and washed twice with distilled water. Cells on the filter paper were dried in an oven at 80 °C until constant weight was achieved and the samples were weighted when cooled to room temperature. 

### 4.2. Mitochondrial Dysfunction

Specific inhibitors with different concentrations, including rotenone (0.01~0.1 mM, SHAM (0.2~2.0 mM), carbonyl cyanide m-chlorophenylhydrazone (CCCP) (0.01~0.1 mM), antimycin A (0.05~0.5 mM), and NaN_3_ (0.01~0.1 mM), were used to induce mitochondrial dysfunction. After 48 h of cultivation, these inhibitors were directly added into the culture. The algal cells were then harvested and used for the subsequent analyses after being continuously cultivated for 36 h.

### 4.3. Pigment Analysis

The lutein and chlorophyll contents were determined following the report by Baroli [65]*.* The wet algal cell pellets obtained after centrifugation (3000× *g*, 5 min) were frozen at *−*70 °C for at least 1 h. The frozen cell pellets were then lyophilized for 36 h in a freeze dryer. Following lyophilization, the dry cell pellets were ground into a powder in a mortar. The pigments were extracted with acetone until the cell debris became colorless. Then the supernatant containing extracted pigments was filtered through a 0.22 μm PTFE membrane (Millipore, Burlington, MA, USA). Each sample (20 μL) was separated on a Waters Spherisorb 5 μm ODS 4.6 mm × 250 mm analytical column (Waters Corp., Milford, MA, USA). Samples were eluted at a flow rate of 1.0 mL/min with a linear gradient from 100% solvent A [acetonitrile: methanol: 0.1 M Tris-buffer, pH 8.0 (84:2:14, by vol.)] to 100% solvent B [methanol: ethyl acetate (68:32, by vol.)] for 15 min, followed by 10 min of solvent B. Individual pigments were identified by comparing their absorption spectra to those of standards (Sigma-Aldrich, St. Louis, MO, USA). The concentration of each pigment was calculated based on the corresponding standard curve. The contents of lutein and chlorophyll were described as the amount of pigments per dry weight (mg/g dry cell weight).

### 4.4. Redox and Energy State Evaluation

The extraction and high-performance liquid chromatography (HPLC) analysis of oxidized nicotinamide adenine dinucleotide (NAD^+^) and reduced nicotinamide adenine dinucleotide (NADH) were conducted based on a previous report [66]. Briefly, the cell pellets were harvested by centrifugation in 20 mL of culture and rinsed with deionized water twice. The cell pellets were then cooled with liquid nitrogen and ground thoroughly in a mortar using a pestle, after which the algal powder was suspended in 10 mL extraction solution containing acetonitrile and 10 mM monopotassium phosphate (KH_2_PO_4_), pH 7.4 (3:1, by volume). The mixture was then homogenized at Ultra-Turrax (Janke & Kunkel, Breisgau, Germany) at 12,000 *g* for 2 min on an ice bath. The supernatant was collected after centrifugation at a speed of 10,000× *g* at 4 °C for 5 min. The pellets were resuspended in 10 mL extraction solution and homogenized again. The supernatant was then combined with the previously obtained supernatant and thoroughly mixed with chloroform. The supernatant, which contained low molecular weight compounds, was collected and stored at −80 °C.

Nucleotides were analyzed following the method of Ganzera et al. [67]. Nucleotides were separated on a Beckman Spherisorb 5 μm ODS C-18 (4.6 mm × 250 mm) analytical column at a flow rate of 1.0 mL/min. Then, 0.1 M KH_2_PO_4_ (pH 6.0) buffer containing 10% methanol was used as the mobile phase to separate the adenosine triphosphate (ATP), adenosine diphosphate (ADP), NAD^+^, and NADH in the sample. An individual peak was identified by comparing the retention time and spectrum with that of the standards. The concentrations of nucleotides in the samples were calculated based on the calibration curves of the freshly prepared standard solutions.

Reduced glutathione (GSH) and oxidized glutathione (GSSG) were analyzed according to Hissin et al. [68]. GSH reacted with *o*-phthalaldehyde (OPT) and produced a highly fluorescent compound in pH 8.0 buffer. The fluorescence intensity was proportional to the concentration of GSH in the sample and could be measured at 420 nm after excitation at 350 nm. GSSG, not GSH, could conjugate with OPT at pH 12.0 and produce a fluorescent compound. The concentrations of GSH and GSSG were calculated using a standard curve.

### 4.5. Protein Extraction

Proteins were extracted from the inhibitor-treated algal cells and the control using a previous method [69]. Briefly, 500 mL of inhibitor-treated or untreated algal cells were harvested by centrifugation (3000× *g*, 5 min) and rinsed with cold deionized water three times. The cell pellets were disrupted by grinding in a mortar after snap-freezing in liquid nitrogen. The sheared cells were then resuspended in extraction solution containing 50 mM Tris-HCl (pH 8.0), 3 mM dithiothreitol (DTT), 5 mM MgCl_2_, 10% glycerol, 0.5% polyvinylpyrrolidone (PVP), 5 mM Na_2_-EDTA, 1 mM phenylmethylsulfonyl fluoride (PMSF), 5 mM benzamidine, and 5 mM aminocaproic acid. The suspension was homogenized for 5 cycles (30 s) at 10,000 g on an ice bath, and the supernatant was collected after centrifugation at 4 °C (10 min, 3000× *g*). Following this, the supernatant was centrifuged at 4 °C at 20,000× *g* for 1 h (Beckman Instruments, Inc., Brea, CA, USA). The proteins in the supernatant were precipitated by following the method developed by Wessel et al. [70]. The obtained protein pellets were washed with pure methanol three times and then air-dried. The dry protein pellets were solubilized with lysis buffer containing 7 M urea, 2 M thiourea, 4% CHAPS, 2% immobilized pH gradient (IPG) buffer (3–10 NL), 0.125 pill/mL protein inhibitor cocktail (complete kit, Roche Diagnostics, Risch-Rotkreuz, Switzerland), 1% nuclease mixture (GE Healthcare, Chicago, IL, USA), and 1.0% DTT. The soluble proteins were precipitated by mixing with nine volumes of cold acetone containing 10% (*w*/*v*) trichloroacetic acid (TCA) and 0.07% (*v*/*v*) 2-mercaptoethanol and maintained at −20 °C overnight. The residual TCA in the protein pellets was removed by thoroughly washing with pre-cooled acetone containing 0.07% 2-mercaptoethanol three times. Residual acetone was removed by air-drying. The protein pellets were resolved with rehydration solution containing 7 M urea, 2 M thiourea, 2% CHAPS, 20 mM DTT, and 0.5% IPG buffer (GE Healthcare). Protein concentration was measured using Coomassie Blue G-250 reagent (Bio-Rad, Richmond, CA, USA) according to the Bradford method [71]. The protein samples were stored at *−*80 °C prior to one-dimensional or two-dimensional electrophoresis.

### 4.6. One-Dimensional SDS Gel Electrophoresis and 2-DE

The one-dimensional SDS electrophoresis was performed using a mini VE electrophoresis apparatus (GE Healthcare, USA). Equal quantities of proteins were loaded into each lane and condensed in 4% stacking gel at 80 V for 1 h and then separated in 12% separating gel for 3 h. 

2-DE was carried out on Amersham Biosciences IPG-phor isoelectric focusing (IEF) system and Hoefer SE 600 (GE Healthcare, Uppsala, Sweden) electrophoresis units following a previous protocol [72]. Briefly, 150 μg protein prepared in 250 μL rehydration solution was loaded into the 13 cm IPG strip (3–10 NL) by rehydration at 30 V for at least 12 h (20 °C). IEF was performed by following a stepwise voltage increasing procedure: 500 and 1000 V for 1 h, respectively, following which the voltage was gradually increased to 8000 V and maintained at 8000 V for about 10 h, resulting in a total of 64,000 Vh. Prior to the two-dimensional electrophoresis, the IPG strips were firstly equilibrated with buffer containing 50 mM Tris-HCl (pH 8.8), 6 M urea, 30% glycerol, 2% SDS, and 1% DTT for 15 min and then incubated with buffer in which 1% DTT was replaced with 2.5% iodoacetamide under darkness for an additional 15 min. Afterwards, the strips were transferred to the upper layer of the 12.5% SDS-PAGE gel, and two-dimensional electrophoresis was carried out at constant 30 mA for about 4 h.

### 4.7. Gel Silver-Staining and Image Analysis

After the two-dimensional electrophoresis, the gels were silver-stained using a previously reported method [72]. In brief, the SDS-PAGE gels were fixed overnight in solution containing 10% acetic acid and 40% ethanol and then incubated in solution containing 0.2% sodium thiosulfate, 4.1% sodium acetate, and 30% ethanol at room temperature for 30 min. The gels were stained in 0.1% silver nitrate solution supplemented with 0.02% formaldehyde for 40 min after washing four times with deionized water. The gels were then incubated with developing solution containing 2.5% sodium carbonate and 0.01% formaldehyde for about 10 min. The development was stopped using EDTA solution (1.46%) and the stained gels were washed with water three times.

Image Scanner equipped with software Lab Scan 3.00 (Amersham Bioscience, San Francisco, CA, USA) was used for the image development of silver-stained gels. Prior to scanning, intensity calibration was conducted using an intensity step wedge. The 2-DE images were analyzed using Image Master 2-D Elite software (Amersham Bioscience, USA). Each protein spot was processed by employing the following procedure: Background subtraction, total spot volume normalization, and calculation of spot volume percentage, which was used for comparing the protein expression level. Only those spots that consistently and significantly deviated at least 1.5-fold compared with the control in at least three experiments were selected for the protein identification.

### 4.8. In-Gel Trypsin Digestion, Mass Spectrometry, and Protein Identification

The spots were excised from the gels and transferred into 0.6 mL centrifuge tubes (AXYGEN, Union City, CA, USA). The gel pieces were then destained by incubation, with solution comprising 1:1 of 30 mM potassium ferricyanide and 100 mM sodium thiosulfate for a few seconds until the dark color disappeared completely. Following this, the gel pieces were washed twice with ultrapure water. The gel chips were equilibrated with 25 mM ammonium bicarbonate (pH 8.0) and then dehydrated with HPLC-grade acetonitrile. The air-dried gel pieces were rehydrated with 2–4 μL trypsin solution [10 μg/mL in 25 mM ammonium bicarbonate (NH_4_HCO_3_)] for 30 min on ice and then covered with 20 μL solution containing 25 mM NH_4_HCO_3_ and 10% acetonitrile. The mixture was then incubated at 37 °C for 16–18 h. Following centrifugation, the residual peptides in the gel pieces were extracted with solution containing 67% acetonitrile and 2.5% trifluoroacetic acid (TFA). The extracted peptide solution was combined with the previous supernatant and concentrated using a freeze-drier. The samples were then directly applied onto the sample plate and combined with an equal volume of matrix solution (5 mg/mL). 

Protocols developed by Ge et al. [73] were used as references. The MALDI-TOF MS and MS/MS spectra were obtained on an ABI 4800 plus MALDI TOF/TOF mass spectrometer (Applied Biosystems, Foster City, CA, USA). The obtained MS and MS/MS data were integrated and interpreted by GPS Explorer software (Applied Biosystems). The combined MS and MS/MS spectra were submitted to the MASCOT search interface (http://www.matrixscience.com/cgi/search_form.pl?FORMVER=2&SEARCH=MIS) (Matrix Science, Ltd., London, UK) using GPS software for database searching. With regards to the search database, the genome database of *Chlorella variabilis* NC64A submitted by the US DOE Joint Genome Institute [74] was set as the first choice. If the identification results were negative, identification by searching against the NCBI Nr (https://www.ncbi.nlm.nih.gov/) and Swiss-Prot databases (https://www.uniprot.org/uniprot/?query=reviewed:yes) or de novo sequencing combined with sequence-similarity searches were also employed. The searching parameters were set as follows: Peptide and MS/MS ion tolerances: 100 ppm and 0.2 Da modifications: Carbamidomethyl and oxidation, one tolerant missed cleavage. A protein identification with a significant (*P* < 0.05) MASCOT protein score was considered to be positive. Identifications with a statistically significant (*P* < 0.05) best ion score based solely on the MS/MS spectra were also accepted as positive. De novo sequencing was carried out on an ABI 4800 plus MALDI TOF/TOF mass spectrometer under the default settings. The peptide sequences were interpreted after filtering out the keratin and trypsin spectra using Mascot Distiller software, with the default settings implemented. The top peptide candidates with the highest score were merged into a single query string, which was submitted to MS-BLAST through the interface at http://genetics.bwh.harvard.edu/msblast/ for searching against the Non-redundant (Nr) database (NRDB95). In sequence similarity searches, the MS-BLAST scoring scheme was used to evaluate the hits. Only high-scoring segment pairs (HSPs) with a score of 62 or above were considered as positive [75].

## 5. Conclusions

Our study confirmed that mRET had a regulatory effect on the biosynthesis of chlorophyll and lutein in *C. pyrenoidosa* under heterotrophic conditions, which is associated with changes in the intracellular redox and energy state. Through proteomics analysis, we identified the related proteins that were important and proposed a network of their roles. These results further our understanding of proteomics of marine natural products, providing valuable insights into future exploration of microalgae and other heterotrophic marine organisms for the enhanced pigment production. 

## Figures and Tables

**Table 1:** Variation in total biomass dry weight (g/L), lutein and chlorophyll contents (mg/g dry cell weight) following mitochondrial dysfunction.

Inhibitors	Biomass (%)	Lutein (%)	Chl. a (%)	Chl. b (%)
Antimycin A (0.1 mM)	59.5 ± 3.2	87.7 ± 3.5	73.2 ± 2.3	73.4 ± 2.5
NaN_3_ (0.05 mM)	68.9 ± 9.6	49.1 ± 1.4	38.9 ± 0.9	45.2 ± 1.7
SHAM (0.5 mM)	46.1 ± 8.6	156.9 ± 7.2	121.6 ± 3.6	136.1 ± 5.4
CCCP (0.032 mM)	42.7 ± 2.6	106.4 ± 3.2	104.5 ± 1.8	128.6 ± 2.8
Rotenone (0.032 mM)	41.4 ± 3.1	107.1 ± 1.6	103.1 ± 1.0	104.9 ± 0.4

The biomass and pigment contents are described as the percentage of the control. Data are expressed as averages ± S.D. of three independent measurements.

**Table 2:** Up- and down-regulated proteins following SHAM, antimycin A and NaN_3_ treatment.

Spot ^a^	Protein Name ^b^	Protein Function and Categorization ^c^	Protein ID ^d^	Species ^e^	MW/*pI*	Peptides ^f^	Score ^g^
**Down-regulated proteins**							
D2	Autophagy-related protein 3	Protein transport, Protein fate (folding, modification, destination)	gi|307105862	*Chlorella vulgaris*	35,252.9/4.42	1	35
D3	Adenylosuccinate synthetase	Purine nucleotide synthesis, Metabolism	gi|307108106	*Chlorella vulgaris*	53,409.7/6.62	3	64
D4	Hypothetical protein CHLNCDRAFT_144859	GMP synthase, Metabolism	gi|307108123	*Chlorella vulgaris*	58,172.5/5.6	8	96
D5	Hypothetical protein CHLNCDRAFT_56182	Adenylosuccinate synthetase, Metabolism	gi|307106668	*Chlorella vulgaris*	64,272.6/5.81	4	59
D6	Phosphoserine aminotransferase	L-glutamate synthesis, Metabolism	gi|307109471	*Chlorella vulgaris*	44,960.5/5.60	1	37
D9	Hypothetical protein CHLNCDRAFT_139931	Alternative splicing factor SRp20/9G8, Transcription	gi|307103428	*Chlorella vulgaris*	19,849.2/11.52	6	53
D10	Hypothetical protein CHLNCDRAFT_134964	SWI-SNF chromatin remodeling complex, Snf 5 subunit, Transcription	gi|307106629	*Chlorella vulgaris*	22,841.5/5.85	1	27
D12	Nitrite reductase	NO biosynthesis, Cell rescue, defense and virulence	gi|116265919	*Chlorella vulgaris*	22,954.7/9.11	1	60
D14	Coproporphyrinogen III oxidase	Key enzyme in heme synthesis, Metabolism	gi|71082810	*Candidatus Pelagibacter ubique*	32,383/9.83	1	68
D15	Hypothetical protein CHLNCDRAFT_30336	ABC transporter superfamily, lipid transport, Protein with binding function or cofactor requirement	gi|307109169	*Chlorella vulgaris*	65,272.95/8.50	1	26
D17	Hypothetical protein CHLNCDRAFT_56437	d-3-phosphoglycerate dehydrogenase, Metabolism	gi|307111670	*Chlorella vulgaris*	63,615.5/6.45	1	41
D19	Nitrite reductase	NO biosynthesis, Cell rescue, defense and virulence	gi|116265919	*Chlorella vulgaris*	22,954.7/9.11	1	69
D21	Malate dehydrogenase	Synthesis of oxaloacetate, Energy	gi|307103202	*Chlorella vulgaris*	35,063.6/8.2	6	104
D22	Hypothetical protein CHLNCDRAFT_57327	Galactokinase activity, Protein with binding function or cofactor requirement	gi|307109337	*Chlorella vulgaris*	55,429.4/5.97	10	66
D25	Phytochrome A	G-protein coupled photoreceptor activity, Transcription	P42500	*Glycine max*	125,653.3/6.16	14	54
D30	Hypothetical protein CHLNCDRAFT_58635	Chaperonin complex component, TCP-1 eta subunit (CCT7), Protein fate (folding, modification, destination)	gi|307105118	*Chlorella variabilis*	61,530.4/6.25	1	28
D36	Hypothetical protein CHLNCDRAFT_56384	UDP-glucose 4-epimerase/UDP-sulfoquinovose synthase, Protein with binding function or cofactor requirement	gi|307103315	*Chlorella variabilis*	39,120.5/6.53	1	77
D37	Hypothetical protein CHLNCDRAFT_49080	Phosphoenolpyruvate carboxykinase activity	gi|307104333	*Chlorella variabilis*	49,051.4/5.69	6	70
D39	Hypothetical protein CHLNCDRAFT_31785	14-3-3 family, multifunctional chaperone, Protein with binding function or cofactor requirement	gi|307106152	*Chlorella variabilis*	29,385.8/4.96	2	77
D40	Hypothetical protein CHLNCDRAFT_34933	Prolyl-tRNA aminoacylation, Protein with binding function or cofactor requirement	gi|307109063	*Chlorella variabilis*	33,163.8/6.56	2	83
D45	Hypothetical protein CHLNCDRAFT_14282	Aconitase/homoaconitase	gi|307110485	*Chlorella variabilis*	11,454.9/5.88	3	53
D46	Expressed protein	Unknown, Unknown	gi|307104059	*Chlorella variabilis*	19,035.7/7.02	1	28
D50	Aspartate carbamoyltransferase	Enzyme of the first committed step in pyrimidine synthesis, Protein activity regulation	P49077	*Arabidopsis thaliana*	43,139.3/6.21	8	55
D51	Hypothetical protein CHLNCDRAFT_35404	Protein serine/threonine kinase, Protein fate (folding, modification, destination)	gi|307107220	*Chlorella variabilis*	40,979.4/9.01	6	56
D53	Hypothetical protein CHLNCDRAFT_52952	Calcium ion binding, Protein with binding function or cofactor requirement	gi|307106250	*Chlorella variabilis*	245,122.6/7.28	1	27
D54	14-3-3-like protein	Multifunctional chaperone, posttranslational modification, Protein with binding function or cofactor requirement	P52908	*Chlamydomonas reinhardtii*	29,495.8/4.9	1	32
D60	Elongation factor 2 (EF-2)	Catalyze GTP dependent ribosomal translocation step during translation elongation, Protein with binding function or cofactor requirement	gi|119167	*Parachlorella kessleri*	94,054.8/5.84	17	308
D65	Expressed protein	Splicing coactivator, RNA processing, Transcription	gi|307109910	*Chlorella variabilis*	84,395.1/10.28	1	28
D69	Citrate synthase	Citrate synthesis, located in mitochondria, Energy	gi|307105555	*Chlorella variabilis*	53,533.7/7.24	5	98
D72	Hypothetical protein CHLNCDRAFT_56456	Dystonin, growth -arrest-specific protein, cytoskeletone, Subcellular localization	gi|307111694	*Chlorella variabilis*	311,562.1/4.77	8	66
D73	Ribosomal protein	Large ribosomal subunit, Protein synthesis	gi|307103203	*Chlorella variabilis*	25,401/8.65	10	138
D80	Hypothetical protein CHLNCDRAFT_138729	Electron transport, Protein with binding function or cofactor requirement	gi|307104457	*Chlorella variabilis*	62,588.1/9.52	11	53
D82	OSJNBb0032E06.9	Ribonuclease H activity, Cell cycle and DNA processing	gi|38344375	*Oryza stiva*	138,142.2/8.96	15	83
D83	Protein W01F3.3a (mlt-11)	Serine-type endopeptidase inhibitor activity, Development	gi|115534910	*Caenorhabditis elegans*	236,516.9/5.07	10	84
D86	Fumarate hydratase	Catalyze S-malate synthesis, mitochondria	gi|17549498	*Ralstonia solanacearum GMI1000*	49,372.3/6.07	4	127
D89	Ribulose bisphosphate carboxylase small chain 1	Carbon dioxide fixation, Energy	P00873	*Chlamydomonas reinhardtii*	20,606.4/9.36	2	66
**Up-regulated proteins**							
U1	Chloroplast thioredoxin peroxidase	Peroxidase activity, Cell rescue, defense and virulence	gi|294845922	*Volvox carteri f. nagariensis*	17,421/5.15	2	193
U2	SMC domain protein	Chromosome structure maintenance, Unknown	gi|296427824	*Heliothis subflexa*	65,238/5.63		87
U3	Peroxiredoxin TSA1	Oxidoreductase, cell redox homeostasis, Cell rescue, defense and virulence	gi|126132194	*Scheffersomyces stipitis CBS 6054*	21,761/4.92	2	82
U4	Magnesium chelatase subunit of protochlorophyllide reductase	Chlorophyll biosynthesis, Protein with binding function or cofactor requirement	gi|254798626	*Parachlorella kessleri*	39,567/5.08	6	310
U5	2-Cys peroxiredoxin	Oxidoreductase, cell redox homeostasis, Cell rescue, defense and virulence	gi|327506370	*Dunaliella viridis*	22,235/5.74	1	51
U6	Hypothetical protein CHLNCDRAFT_48133	Ornithine carbamoyltransferase, Protein with binding function or cofactor requirement	gi|307109894	*Chlorella variabilis*	38,764.5/6.68	9	51
U7	Hypothetical protein CHLNCDRAFT_53139	Antioxidant activity, Cell rescue, defense and virulence	gi|307106076	*Chlorella variabilis*	21,778.9/8.35	1	40
U8	Hypothetical protein BAL199_15803	Unknown, Unknown	gi|163792326	*alpha proteobacterium*	42,102.3/5.49	12	88
U9	Hypothetical protein CHLNCDRAFT_48477	Membrane transport, Cellular transport, transport facilitation and transport routes	gi|307110872	*Chlorella vulgaris*	31,637.3/9.49	1	29
U10	tRNA(Ile)-lysidine synthase, chloroplastic	Ligase activity, translation, Transcription	Q32RX0	*Staurastrum punctulatum*	58,797/10.16	1	29
U11	Penecillin-binding protein 2	Penicillin binding, Protein with binding function or cofactor requirement	gi|163752395	*Shewanella benthica*	68,820.33/9.54		67
U12	Aldehyde dehydrogenase	Oxidation of aldehyde, Metabolism	gi|285018869	*Xanthomonas albilineans*	54,004.7/6.05		64
U13	Hypothetical protein CHLNCDRAFT_30965	Structural constitute of ribosome, Protein synthesis	gi|307107744	*Chlorella vulgaris*	21,281.2/10.33	8	51
U14	Hypothetical protein CHLNCDRAFT_143237	Dystonin, growth arrest specific protein, Subcelluar location	gi|307109339	*Chlorella vulgaris*	56,981.7/5.56	13	53
U15	FG-GAP repeat protein	Ligand binding, Unknown	gi|40062562	*Uncultureed marine bacterium 159*	136,477.9/4.18		66
U16	Expressed protein	Unknown, Unknown	gi|307111048	*Chlorella variabilis*	20,888.8/10.28	6	57
U17	Hypothetical protein CHLNCDRAFT_59525	nuclear receptor binding factor-1, Cell rescue, defense and virulence	gi|307111650	*Chlorella variabilis*	34,897.1/5.06		68
U18	Hypothetical protein PEPMIC_01485	Unknown, Unknown	gi|160947550	*Peptostreptococcus micros*	18,170.3/4.67	7	83
U19	Hypothetical protein CHLNCDRAFT_58231	Ribosomal protein L22, Protein synthesis	gi|307105888	*Chlorella vulgaris*	67,650.2/10.05	1	29
U20	C protein alpha-antigen	Receptor, Protein with binding function or cofactor requirement	gi|307708369	*Streptococcus mitis NCTC 1226*	34,6246.9/4.98		65
U21	4-hydroxy-3-methylbut-2-en-1-yl diphosphate synthase	Oxidoreductase, terpenoids biosynthesis, Protein with binding function or cofactor requirement	Q5QYA9	*Idiomarina loihiensis*	40,462/5.68	1	58
U22	N-(5’-phosphoribosyl)anthranilate isomerase	Tryptophan biosynthesis, Metabolism	gi|307107003	*Chlorella vulgaris*	20,906.8/5.55	1	31
U23	Hypothetical protein CHLNCDRAFT_37743	Aldehyde dehydrogenase, Metabolism	gi|307102335	*Chlorella variabilis*	45,285.3/6.11	3	111
U24	Hypothetical protein OsI_036678	Calcium ion binding, Protein with binding function or cofactor requirement	gi|125536231	*Oryza sativa*	31,743.5/9.98	10	93
U25	Chloroplast 30S ribosomal protein S4	Structural constituent of ribosome, Protein synthesis	P59137	*Catharomnion ciliatum*	23,589.9/10.3	1	30
U26	Hypothetical protein CHLNCDRAFT_140182	Transcription initiation factor, Transcription	gi|307103188	*Chlorella variabilis*	63,894.8/5.19	13	54
U27	Nitrate reductase [NADH] 1	Catalyze nitrite synthesis, Metabolism	P16081	*Oryza sativa*	101,447.9/6.19	1	29
U28	Ribulose-1,5-bisphosphate carboxylase/oxygenase large subunit	Carbon fixation, Energy	gi|164455027	*Chlorella variabilis*	52,496.3/5.99	1	32
U29	Hypothetical protein MGG_08723	Unknown, Unknown	gi|145601241	*Magnaporthe grisea*	36,967.8/5.69	8	83
U30	Expressed protein	Large subunit of ribosome, Protein synthesis	gi|307108236	*Chlorella variabilis*	9805.4/11.71	4	69
U31	Hypothetical protein CHLNCDRAFT_59740	3-oxoacyl-(acyl-carrier-protein) synthase, lipid transport, Cellular transport, transport facilitation and transport routes	gi|307104988	*Chlorella variabilis*	32,230.3/6.19	1	28
U32	Hypothetical protein CHLNCDRAFT_18194	Unknown, Unknown	gi|307111928	*Chlorella variabilis*	9084.4/4.37	1	28
U33	Ribulose-1,5-bisphosphate carboxylase/oxygenase large subunit	Carbon fixation, Energy	gi|164455037	*Chlorella variabilis*	52,496.3/6.0	18	193
U34	Glucose-6-phosphate isomerase	Isomerase, involved in glycolysis, Energy	gi|284434863	*Parachlorella kessleri*	27,035/5.35	1	48
U35	Hypothetical protein CHLNCDRAFT_143799	Unknown, Unknown	gi|307108818	*Chlorella variabilis*	43,154.4/9.57	1	27
U36	Superoxide dismutase	Antioxidant enzyme, Cellular communication/signal transduction mechanism	gi|34558145	*Wolinella succinogenes DSM 1740*	25,795.1/8.89		78
U37	Hypothetical protein CHLNCDRAFT_138879	FAP-dependent helicase activity, Transcription	gi|307104244	*Chlorella variabilis*	59,382.6/9.77	1	27
U38	Glucose -6-phosphate isomerase	Glycolysis enzyme, Energy	gi|307105594	*Chlorella variabilis*	72,048.5/6.41	7	53
U39	GTP-binding protein	Intracellular protein transport, Cellular communication/signal transduction mechanism	gi|307106020	*Chlorella variabilis*	25,468.9/6.66	2	66

All the down-regulated protein spots were labeled as Dn. The up-regulated protein spots were labeled as Un. Down-regulation means a decreased protein expression when the test group (after inhibitor treatment) was compared to the control group. Up-regulation means an increased protein expression when the test group was compared to the control group. ^a^ refers to the number in Figure 4. ^b^ protein name from NCBI Nr and Swiss-prot database. ^c^ function of hypothetical proteins is described according to the GO (gene ontology) information. ^d^ accession number from NCBI Nr database or Swiss-prot database. ^e^ species name from NCBI Nr and Swiss-prot database. ^f^ number of peptides used for the protein identification, those proteins with no peptides number listed were identified through de novo sequencing and MS-BLAST search against nrdb95 database. ^g^ score is defined as −10 × Log (P), where P is the absolute possibility and is closely related the size of database used for searching and automatically calculated by the MASCOT search engine. The minimal threshold value was 25. For the MS-Blast identification, the score meant the HSPs. MW, molecular weight.

**Table 3:** Variation in F.D. of up-regulated and down-regulated proteins following SHAM, antimycin A and NaN_3_ treatment.

Spot ^a^	F.D. ^h^		
	SHAM	Antimycin A	NaN_3_
Down-regulated proteins			
D2	−4.95 ± 0.58	−5.27 ± 2.16	
D3	−6.57 ± 0.59		
D4	−3.01 ± 0.11	−2.37 ± 0.22	−2.29 ± 0.86
D5	−2.22 ± 0.30		−1.93 ± 0.20
D6	−2.18 ± 0.11		
D9	−1.81 ± 0.35		
D10	−1.77 ± 0.35		
D12	−2.37 ± 0.74	−3.12 ± 0.05	
D14	−1.95 ± 0.20	−1.98 ± 0.43	−2.29 ± 0.59
D15	−2.27 ± 0.66	−6.95 ± 2.48	
D17	−2.45 ± 0.98	−4.46 ± 1.59	−3.87 ± 0.79
D19	−2.42 ± 0.98	−2.08 ± 0.25	
D21	−2.26 ± 0.82		
D22	−1.87 ± 0.26	−6.32 ± 1.36	−1.87 ± 0.26
D25		>−100	>−100
D30		>−100	>−100
D36		−3.03 ± 1.22	−3.08 ± 0.91
D37		−3.32 ± 0.65	−5.66 ± 0.04
D39		−2.27 ± 0.57	
D40		>−100	>−100
D45		−2.03 ± 0.02	
D46		−1.77 ± 0.37	
D50		−1.98 ± 0.14	
D51		−1.65 ± 0.24	
D53		−1.89 ± 0.46	−1.89 ± 0.46
D54		−1.85 ± 0.33	
D60			>−100
D65			>−100
D69			−3.55 ± 0.81
D72			−2.54 ± 0.97
D73			−4.20 ± 1.37
D80			−2.24 ± 0.52
D82			−1.89 ± 0.25
D83			−1.89 ± 0.30
D86			−1.59 ± 0.20
D89			−1.76 ± 0.38
Up-regulated proteins			
U1	1.65 ± 0.57		
U2	1.72 ± 0.48		
U3	1.53 ± 0.16	1.62 ± 0.08	
U4	1.60 ± 0.09		
U5	1.84 ± 0.37		
U6	1.92 ± 0.46		
U7	1.85 ± 0.33		
U8	1.65 ± 0.57	1.59 ± 0.28	1.94 ± 0.68
U9	1.54 ± 0.18		
U10	2.13 ± 0.37	2.75 ± 0.02	
U11	1.68 ± 0.30		
U12	2.53 ± 0.78		
U13	2.56 ± 0.73		3.96 ± 2.05
U14	2.18 ± 0.10		
U15	3.08 ± 1.38		
U16	2.66 ± 0.45		
U17	2.21 ± 0.25		
U18	3.46 ± 0.93		
U19	4.38 ± 1.02		
U20	4.05 ± 0.09		
U21	5.36 ± 0.05	3.75 ± 0.01	1.53 ± 0.21
U22	6.89 ± 1.10	3.61 ± 0.86	
U23	>100.00	5.39 ± 0.69	
U24	>100.00		
U25		1.79 ± 0.53	1.89 ± 0.39
U26		1.77 ± 0.39	
U27		1.60 ± 0.05	
U28		1.92 ± 0.48	
U29		1.92 ± 0.44	
U30		2.36 ± 0.71	
U31		2.56 ± 0.75	
U32		2.16 ± 1.08	
U33		3.02 ± 0.08	
U34		>100	
U35			1.75 ± 0.21
U36			1.60 ± 0.03
U37			2.33 ± 0.20
U38			>100
U39			3.75 ± 0.01

All the down-regulated protein spots were labeled ash Dn. The up-regulated protein spots were labeled as Un. ^a^ refers to the number in Figure 4. ^h^ fold difference (F.D.) is used to describe the protein expression level variation following inhibitors treatment compared with the control and expressed as mean ± S.D. from three independent experiments. Positive values mean up-regulation and negative values mean down-regulation.

## Supplementary Materials

The following are available online at http://www.mdpi.com/1660-3397/16/10/354/s1, Figure S1: Functional categorization of identified up- and down-regulated proteins in *Chlorella pyrenoidosa* after treatment with mitochondrial respiratory electron transport chain (mRET) inhibitors. A: SHAM treatment. B: antimycin A treatment. C: NaN_3_ treatment. This classification is based on homologies.
