# Supplementary material for: Role of Mitochondria in Regulating Lutein and Chlorophyll Biosynthesis in Chlorella pyrenoidosa under Heterotrophic Conditions
Source: Mar Drugs. 2018 Sep 28;16(10):354. doi: 10.3390/md16100354 (PMC6213193; doi:10.3390/md16100354)
Supplement: Supplementary file 1 [file marinedrugs-16-00354-s001.pdf]

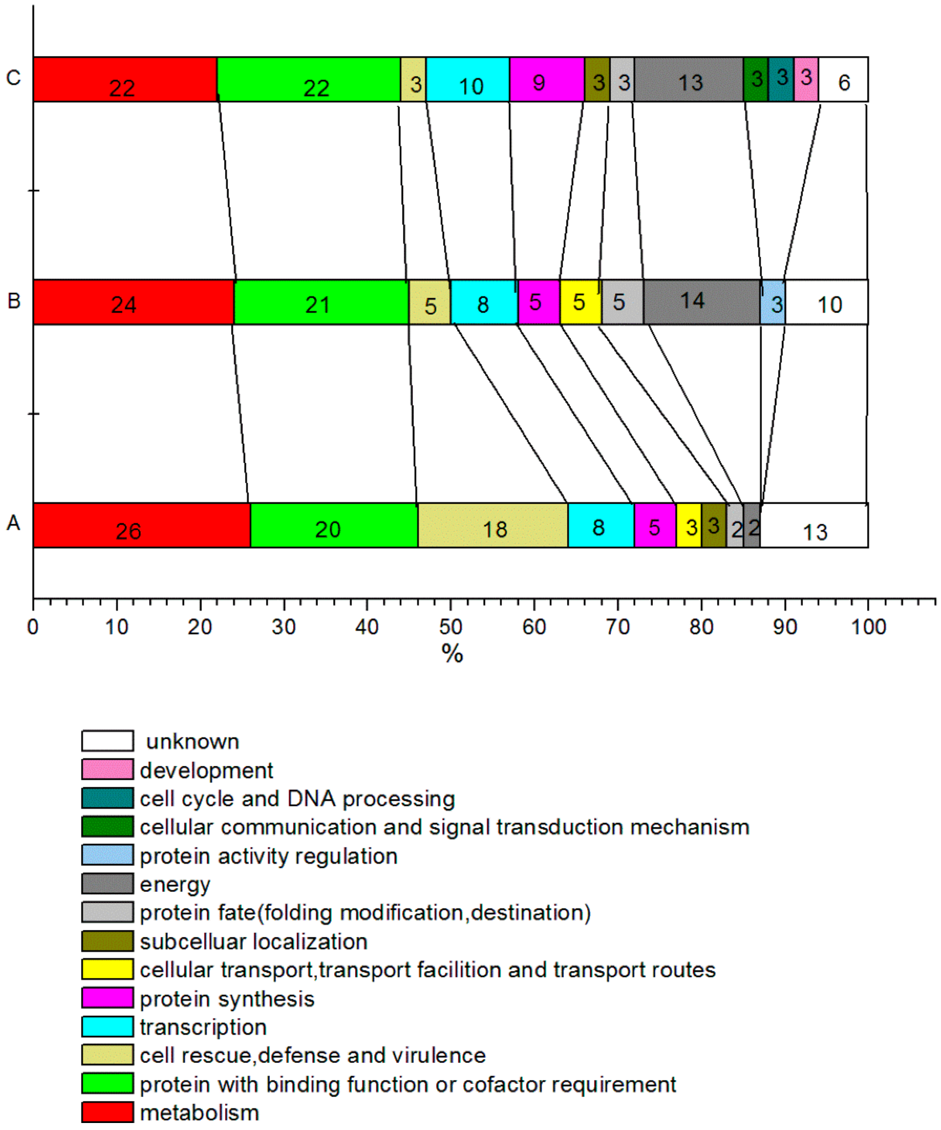


**Figure S1.** Functional categorization of identified up- and down-regulated proteins in *Chlorella pyrenoidosa* after treatment with mitochondrial respiratory electron transport chain (mRET) inhibitors. (**A**) Salicylhydroxamic acid (SHAM) treatment; (**B**) antimycin A treatment; (**C**) sodium azide ($NaN_3$) treatment. This classification is based on homologies.
